# Metabolism and Intracranial Epileptogenicity in Temporal Lobe Long-Term Epilepsy-Associated Tumor

**DOI:** 10.3390/jcm11185309

**Published:** 2022-09-09

**Authors:** Jiajie Mo, Jianguo Zhang, Wenhan Hu, Lin Sang, Xiaoqiu Shao, Chao Zhang, Kai Zhang

**Affiliations:** 1 Department of Neurosurgery, Beijing Tiantan Hospital, Capital Medical University, Beijing 100070, China; 2 Department of Neurosurgery, Beijing Neurosurgical Institute, Capital Medical University, Beijing 100070, China; 3 Department of Neurosurgery, Beijing Fengtai Hospital, Beijing 100070, China; 4 Department of Neurology, Beijing Tiantan Hospital, Capital Medical University, Beijing 100070, China

**Keywords:** LEAT, temporal lobe epilepsy, metabolism, epileptogenicity

## Abstract

Brain tumors are common in epilepsy surgery and frequently occur in the temporal lobe, but the optimal surgical strategies to remove the tumor and epileptogenic zone remain controversial. We aim at illustrating the positron emission tomography (PET) metabolism and the stereoelectroencephalography (SEEG) epileptogenicity of temporal lobe long-term epilepsy-associated tumors (LEAT). In this study, 70 patients and 25 healthy controls were included. Our analysis leveraged group-level analysis to reveal the whole-brain metabolic pattern of temporal lobe LEATs. The SEEG-based epileptogenicity mapping was performed to verify the PET findings in the epileptic network. Compared to controls, patients with a temporal lobe LEAT showed a more widespread epileptic network based on ^18^FDG-PET in patients with a mesial temporal lobe LEAT than in those with a lateral temporal lobe LEAT. The significant brain clusters mainly involved the paracentral lobule (ANOVA *F* = 9.731, *p* < 0.001), caudate nucleus (ANOVA *F* = 20.749, *p* < 0.001), putamen (Kruskal–Wallis *H* = 19.258, *p* < 0.001), and thalamus (ANOVA *F* = 4.754, *p* = 0.011). Subgroup analysis and SEEG-based epileptogenicity mapping are similar to the metabolic pattern. Our findings demonstrate the metabolic and electrophysiological organization of the temporal lobe LEAT epileptic network, which may assist in a patient-specific surgical strategy.

## 1. Introduction

Brain tumors are the second most common histopathological diagnosis in epilepsy surgery, accounting for 23.6% of all cases [1]. Such brain tumors are defined as low-grade, developmental, long-term epilepsy-associated tumors (LEATs), with the following characteristics: (1) 77% of cases occur in the temporal lobe; (2) most LEATs are of low-grade malignancy and classified as World Health Organization (WHO) I°; and (3) LEATs are often composed of mixed glial and neuronal cell components and present with variable growth patterns, including small cysts or nodules. LEAT entities continue to be discovered and are comprise of the ganglioglioma (GG), the dysembryoplastic neuroepithelial tumor (DNT), the pilocytic astrocytoma (PA), low-grade neuroepithelial tumors (LGNETs, not otherwise specified), the isomorphic diffuse glioma (IDG), the angiocentric glioma (AG), multinodular and vacuolated neuronal tumors of the cerebrum (MVNT), and the papillary glio-neuronal tumor (PGNT) [2].

Though epilepsy surgery is reported as the effective approach for LEATs [3], with 70–90% of patients achieving the long-term seizure-free condition [4,5], the optimal individual resective strategy remains inconclusive. Anterior temporal lobectomy (ATL) was a well-established surgical procedure to treat mesial temporal lobe epilepsy (mTLE), associated with hippocampal sclerosis (HS) [6]. Whether or not patients with mesial structure LEATs are indicated to undergo the same ATL is debated, as temporo-mesial LEATs have a widespread epileptic network and complex epileptogenic mechanisms. To avoid the risks of cognitive and lingual deficits associated with ATL, some researchers have sought replaceable and minimally resective strategies, such as selective amygdalohippocampectomy [7], parahippocampectomy [8], and hippocampal sparing temporal lobectomy [9,10,11], to achieve the same prognosis with decreased functional impairment. Furthermore, the best surgical strategies to estimate the epileptogenic zone and the minimal area to remove temporal neocortex LEATs are still debated. These dilemmas represent contemporary issues in epilepsy surgery [12,13].

Interictal positron emission tomography (PET) with fluorodeoxyglucose (^18^F-FDG) has largely proved its utility in the presurgical evaluation of drug-resistant epilepsies (DRE). Its hypometabolism range is conducive to indicating the functional deficit zone, as well as to delineating the epileptic network [13]. It is reported that interictal ^18^F-FDG PET metabolic patterns have shown satisfactory correspondence among distinct subtypes of clinical TLE, with specific patterns in scalp electroencephalography (EEG) or intracranial EEG (iEEG) [14]. In clinical practice, the iEEG technique mainly includes stereoelectroencephalography (SEEG) and an electrocorticogram (ECoG). It is recommended when noninvasive evaluations fail to determine EZ [15]. It helps us better determine the neuronal networks involved in the seizure onset zone (SOZ) and spread areas and guides the surgical resection. Calculation of the epileptogenicity index (EI) has been proposed recently to represent the likelihood of various regions being part of the seizure onset zone [16]. The quantification of the seizure onset zone and mapping multi-channels to a time-series of statistical parametric maps help to provide better pre-surgical assessment and support clinical decisions. Clinically, SEEG implantation is uncommon in tumor-related epilepsy surgery, as the border of the tumor is usually explicit for direct resection, which makes the intracranial electrophysiological data of tumors rare. SEEG is considerable only in some cases with unclear anatomo-electroclinical or functional area involvement to establish an individual surgical strategy.

Brain tumors are the second most common pathology in epilepsy surgery and are mainly located in the temporal lobe [1]. The tumor location may affect the brain network and clinical surgical strategy. To date, the optimal surgical strategies to accurately remove the tumor and epileptogenic zone for patients with tumor-associated temporal lobe epilepsy remain controversial. We propose that Lesionectomy is recommended for lateral temporal lobe LEATs, while an extended surgical range is suitable for medial temporal lobe LEATs. Therefore, the present study aims at illustrating the PET metabolic pattern and the SEEG epileptogenicity mapping of different subtypes of temporal lobe LEATs. The results may assist in a patient-specific surgical strategy.

## 2. Materials and Methods

### 2.1. Patient Selection

**Figure 1:**
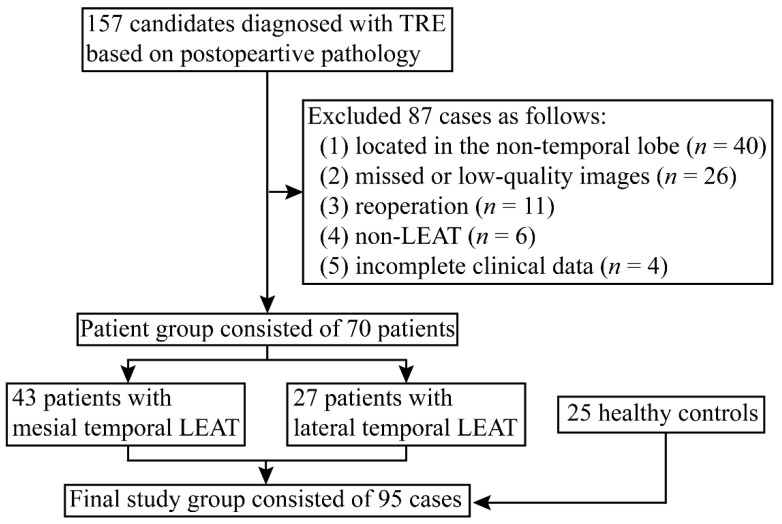
Flowchart of 157 consecutive patients diagnosed with tumor-related epilepsy. Eighty-seven patients were excluded due to exclusion criteria. Finally, this study consisted of 70 patients and 25 healthy controls. TRE: tumor-related epilepsy; LEAT: long-term epilepsy-associated tumor.

This study was approved by the Ethics Committee of the Beijing Tiantan Hospital (KYSQ 2021-366-01), and written informed consent was obtained from all participants. Between January 2015 and December 2020, 157 consecutive participants diagnosed with tumor-related epilepsy (TRE), based on postoperative pathology, were enrolled. All of them had the accordant anatomical–electroclinical correlations in presurgical evaluation, and 87 patients were excluded as: (1) the tumor was not located in the temporal lobe; (2) there was missed or low-quality neuroimaging; (3) there was reoperation; (4) the result was not a LEAT; and (5) there was incomplete clinical data. We then divided the 70 included patients into mesial temporal LEAT and lateral temporal LEAT groups. Twenty-five healthy controls were included for the purpose of metabolic pattern analysis (Figure 1).

### 2.2. Neuroimaging Acquisition

All included participants underwent the same structural and metabolic neuroimaging protocols, similar to our previous works [17,18]. Using a 3T Siemens Verio scanner, structural MRI scans were acquired for all participants, including the T_1_WI MPRAGE sequence (repetition time [TR] = 2300 ms, echo time [TE] = 2.53 ms, flip angle = 12°, slice thickness = 1 mm, no gap, voxel size = 1.0 mm × 1.0 mm × 1.0 mm) and the T_2_WI FLAIR sequence (TR = 7000 ms, TE = 80 ms, flip angle = 12°, slice thickness = 1 mm, no gap, voxel size = 1.5 mm × 1.5 mm × 1.5 mm). Interictal ^18^FDG-PET examinations were performed under standard resting conditions using the GE Discovery ST PET-CT system (300 mm FOV, matrix 192 × 192, 3.27 mm slice thickness). Patients were required to rest quietly in a dimly lit room during the 40 min following the intravenous administration of ^18^F-FDG at a mean dose of 310 MBq/70 kg body weight. The ordered subset expectation maximization (OSEM) algorithm (16 subsets and 6 iterations) was used for PET data reconstruction. Reconstructed images were corrected for attenuation using transmission scans obtained from a germanium source. PET scans of all patients were obtained within 6 months before epilepsy surgery evaluation. None of the patients had clinical seizures in the 6 h before or during the PET scan.

### 2.3. Metabolic Pattern Analysis

PET scans were analyzed with standalone SPM12 [19] on MATLAB 2021a software (MathWorks, Natick, MA, USA). The image preprocessing steps comprised of co-registration to structural images, spatial normalization to the MNI template, proportional scaling intensity normalization, and smoothing using a Gaussian kernel, with a full width at half maximum (FWHM) of 8 × 8 × 8 mm^3^. Afterward, PET images of the left temporal LEATs had to be left–right flipped for further statistical analysis to ensure a homogeneous group, with the lesion focus on the same side in all patients.

For the group comparison, voxel-wise Student’s *t*-test separately compared the whole-brain PET value between patients with mesial and lateral temporal lobe LEATs and controls. To address possible confounds of clinical information, we selected age and sex as confounding covariates. Considering the variability of tumor shape and location, we further divided the mesial temporal lobe LEAT group into amygdala–hippocampal LEAT and temporal base LEAT subgroups and repeated the analysis. Corrections in multiple comparisons were performed using the random field theory (RFT)-based family-wise error (FWE) correction. A two-sided *p* value <0.05 was considered to indicate statistical significance. Cluster significance thresholds (extent threshold) were set at 100 contiguous voxels to reduce type I errors introduced by potential noise. According to the significant cluster findings, regions-of-interest (ROI)-based analysis was further performed. In the present study, paracentral lobule (PCL), caudate nucleus (CN), putamen, and thalamus were selected as ROI and compared across groups.

### 2.4. SEEG Recording

After the multidisciplinary evaluation, two patients were needed to undergo SEEG implantation to further determine the origin of the seizure and confirm the resective range. They were implanted with clinical depth electrodes (Huake-Hengsheng Medical Technology, Beijing, China). Clinical depth intracranial EEG (iEEG) was recorded using a clinical long-term monitoring system (Neurofax EEG-1200, 128 or 256 channels, Nihon Kohden, Tokyo, Japan), with a sampling rate of 1000 or 2000 Hz and a bandpass filter, with a frequency response range of 1.6–300 or 0.08–600 Hz. Each depth electrode had a diameter of 0.8 mm with 8–16 contacts, and the inter-contact spacing was 3.5 mm (center to center). The clinical decision for electrode placement was based on presurgical non-invasive information, which provided hypotheses about the location of the epileptogenic zone. The depth electrodes were placed using a CRW frame-based system (Integra Radionics, Burlington, MA, USA) and a predefined path. Postoperative computed tomography (CT) was performed to confirm the absence of intracranial bleeding and the accuracy of the electrode positioning. Long-term SEEG monitoring was performed 24 h after electrode implantation to record at least two habitual seizures. To reduce distortion during the recording, iEEG signals were referenced to the most electrographically silent channel outside the seizure focus (typically a white matter channel).

### 2.5. Epileptogenicity Mapping

A baseline void of any epileptic activity or artifact was chosen during non-rapid eye movement sleep recording. All seizure episodes recorded by video monitoring and ictal SEEG patterns of electrical onset were identified visually during presurgical evaluation. The analysis was performed in a bipolar montage. Any subclinical electric patterns were excluded. Epileptiform activity during and between seizures was recorded and analyzed using epileptogenicity mapping (EM) [20]. This methodology was designed to identify the brain regions likely to produce significantly greater high-frequency activity (between 80 and 250 Hz) than at baseline using standard techniques from imaging time-series analysis, which involved a simple categorical comparison (two-sample *t* test) of the mean activity at baseline (30 s) and mean activity over sliding windows (5 s) after seizure onset. EM for each patient was conducted after local interpolation of the *t* value to produce an image superimposed on the individual MRI

### 2.6. Statistical Analysis

After confirming the normality of data distribution (Lilliefors test) and homogeneity of variances (Levene test), continuous variables were compared using Student’s *t*-test or the Mann–Whitney *U* test for 2 independent samples, one-way analysis of variance (ANOVA), and post *hoc* pairwise test with multiple comparison correction for multiple samples. The distribution of categorical variables was compared using the Chi-squared (*χ^2^*) test. All statistical analyses were performed with SPSS software version 26.0 (Armonk, NY: IBM Corp). Two-sided, *p* < 0.05 was considered indicative of a significant difference.

## 3. Results

### 3.1. Patients’ Characteristics

This study group consisted of 70 patients diagnosed with temporal lobe LEATs (43 patients with a mesial temporal LEAT and 27 with a lateral temporal LEAT). Thirty-four patients (48.6%) were female, and epileptogenic lesions of 29 patients (41.4%) were located on the left side. The mean age of onset, duration, and age of surgery was 15.1 ([standard deviation, SD] = 10.6), 6.4 (6.5), and 21.6 (11.6) years, respectively. The mean follow-up was 43.8 (15.6) months. Of 70 patients with a temporal lobe LEAT, 53 (75.7%) had GG, 10 (14.3%) had DNT, 3 (4.3%) had LGNETs, 2 (2.9%) had AG, and 1 (1.4%) had PA and PGNT.

A total of 25 healthy controls were included in the PET group analysis, with no significant differences in age or sex compared to the patient group (sex: Pearson *χ^2^* = 0.192, *df* = 2, *p* = 0.909; age: ANOVA *F* = 0.2.964, *df* = 2, *p* = 0.057).

Details of clinical information are shown in Table 1.

### 3.2. Mapping Metabolic Alteration to Controls

Metabolic analysis was performed on the 70 patients with a temporal lobe LEAT and compared with the 25 age- and sex-match healthy controls.

**Figure 2:**
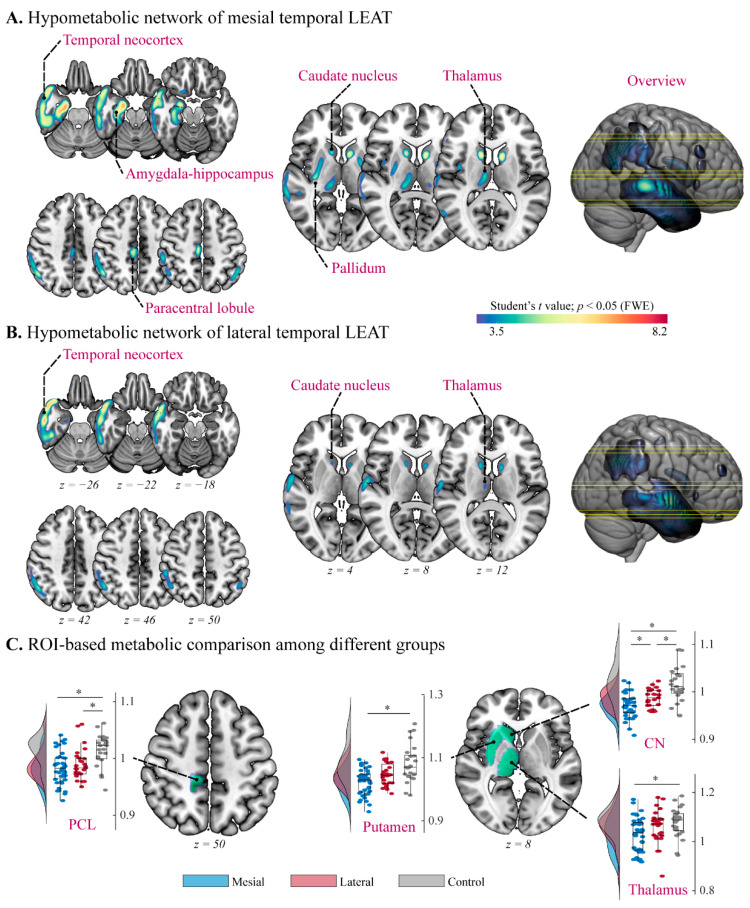
Group-level alterations in metabolism in patients with temporal lobe long-term epilepsy-associated tumors (LEAT). (**A**,**B**) Voxel-based *t* statistical parametrical map demonstrated the hypometabolic networks of mesial and lateral temporal LEATs, respectively. FWE: family-wise error. (**C**) Within-group comparison of the region of interest (ROI)-based metabolic value derived from significant group comparison. *: *p* < 0.05 (Bonferroni correction); PCL: paracentral lobule; CN: caudate nucleus; PET values among groups are expressed in boxplot as mean and standard deviation (SD).

In the mesial temporal lobe group (*n* = 43), compared to controls, the metabolic network showed widespread involvement in mesial temporal structures, temporal neocortex, and subcortical structures (Figure 2A), suggesting hypometabolism in the amygdala–hippocampus complexity (voxel peak coordinates [*x, y, z*] = 20, −9, 24 with *t* = 7.00), temporal neocortex (*x* = 46, *y* = 16, *z* = −27 with *t* = 5.62), PCL (*x* = 8, *y* = −26, *z* = 48 with *t* = 5.02), caudate nucleus (*x* = 14, *y* = 9, *z* = 15 with *t* = 6.20), pallidum (*x* = 32, *y* = −16, *z* = 0 with *t* = 5.05), and thalamus (*x* = 9, *y* = −16, *z* = 12 with *t* = 4.63).

In the lateral temporal lobe group (*n* = 27), compared to controls, the metabolic network displayed relatively limited involvement in temporal neocortex and the subcortical structures (Figure 2B), suggesting hypometabolism in the temporal neocortex (*x* = 60, *y* = −3, *z* = −9 with *t* = 6.44), caudate nucleus (*x* = 14, *y* = 9, *z* = 15 with *t* = 4.76), and thalamus (*x* = 8, *y* = −14, *z* = 12 with *t* = 3.85).

The ROI-based analysis indicated the hypometabolism of PCL in both patient groups (ANOVA *F* = 9.731, *p* < 0.001; mesial vs. control: mean difference = −0.031, *p* < 0.001; lateral vs. control: mean difference = −0.024, *p* = 0.010), the hypometabolism of putamen (Kruskal–Wallis *H* = 19.258, *p* < 0.001; mesial vs. control: mean difference = −30.207, *p* < 0.001) and thalamus (ANOVA *F* = 4.754, *p* = 0.011; mesial vs. control: mean difference = −0.049, *p* = 0.017) only in the mesial temporal LEAT group, and the significant differences of caudate nucleus (ANOVA *F* = 20.749, *p* < 0.001; mesial vs. lateral: mean difference = −0.015, *p* = 0.017; mesial vs. control: mean difference = −0.045, *p* < 0.001; lateral vs. control: mean difference = −0.030, *p* = 0.004) among three groups (Table 2).

### 3.3. Subgroup Analysis of Metabolic Alteration

Considering the variability of temporal neocortex tumors, we further divided the mesial temporal LEAT group into the amygdala–hippocampal LEAT group and the temporal base LEAT group (two cases were excluded as the tumor involved above structures) and compared them to controls, respectively.

**Figure 3:**
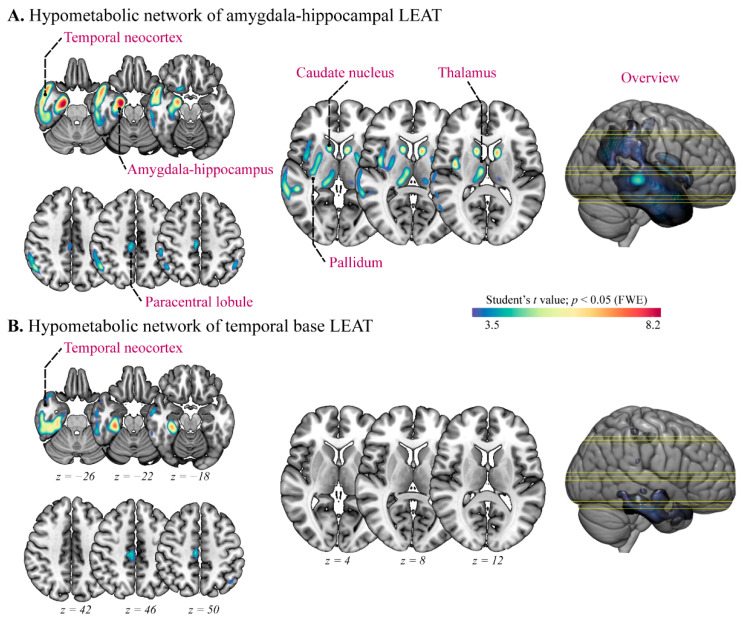
Subgroup analysis of metabolic alteration in mesial temporal lobe long-term epilepsy-associated tumors (LEAT). (**A**,**B**) Voxel-based *t* statistical parametrical map demonstrated the widespread hypometabolic networks of amygdala–hippocampal and temporal base LEAT groups, respectively. FWE: family-wise error.

Compared to controls, the amygdala–hippocampal LEAT group (*n* = 18) showed a widespread and significant hypometabolic pattern, similar to the mesial temporal lobe LEAT group (Figure 3A), including amygdala–hippocampus complexity (*x* = 21, *y* = −10, *z* = −24 with *t* = 8.99), temporal neocortex (*x* = 36, *y* = 8, *z* = −39 with *t* = 8.43), PCL (*x* = 2, *y* = −30, *z* = 34 with *t* = 5.29), caudate nucleus (*x* = −15, *y* = −27, *z* = 8 with *t* = 4.14), pallidum (*x* = 30, *y* = −14, *z* = −4 with *t* = 5.30), and thalamus (*x* = 8, *y* = −15, *z* = 10 with *t* = 5.56). However, the temporal base LEAT group (*n* = 13) represented the limited significant results in the temporal base (*x* = 30, *y* = −30, *z* = −22 with *t* = 7.47) (Figure 3B).

### 3.4. Epileptogenicity Mapping of Temporal Lobe LEAT

In presurgical evaluation, two patients had no indicative anatomical-electroclinical correlation to guide the epilepsy surgery and then underwent SEEG implantation and long-term monitoring.

**Figure 4:**
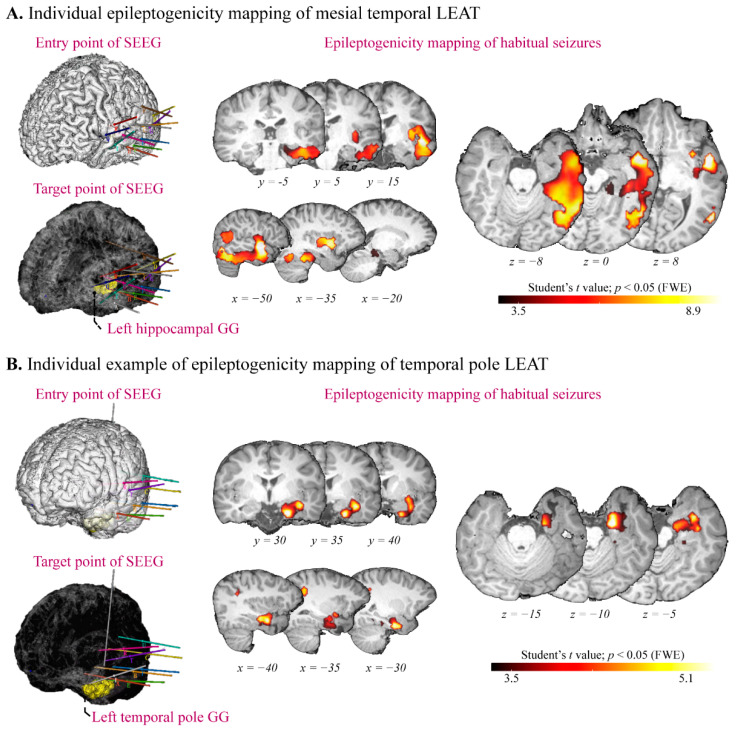
Epileptogenicity mapping of temporal lobe long-term epilepsy-associated tumors (LEAT). Representation of The SEEG implantation strategy, lesion location (left panel), and the epileptogenicity mapping (right panel) of (**A**) patient 64, with hippocampal ganglioglioma (GG) and (**B**) Patient 70, with temporal pole GG. FWE: family-wise error.

Patient 64, with hippocampal GG, was an 11-year-old, right-handed boy. The visual analysis of MRI/^18^FDG-PET registration revealed widespread hypometabolism in the left temporal lobe and the insular lobe. The patient underwent SEEG because of suspected “temporal lobe epilepsy plus.” SEEG implantation covered the anterior temporal lobe and the insular lobe. SEEG indicated the origin in the hippocampus and the adjacent cortex. The patient finally underwent extended lesionectomy and remained seizure-free for 26 months without complications. Retrospective epileptogenicity mapping results show the widespread range in the hippocampal (*x* = −48, *y* = 26, *z* = 11 with *t* = 10.75), temporal neocortex (*x* = −57, *y* = −31, *z* = 11 with *t* = 9.83), and insular lobes (*x* = −39, *y* = 26, *z* = 14 with *t* = 8.65) (Figure 4A).

Patient 70, with temporal pole GG, was a 13-year-old, right-handed girl. Seizure symptomatology included right-hand numbness, which could not exclude the functional area or the insular lobe being involved. Therefore, the patient underwent SEEG and the implanted electrodes covered the temporal and insular lobes. SEEG indicated the origin in the perilesional cortex. The patient finally underwent tailored temporal resection and remained seizure-free for 27 months without complications. Retrospective epileptogenicity mapping results show the limited significant results in the perilesional cortex (*x* = −27, *y* = 26, *z* = −13 with *t* = 4.26) (Figure 4B).

This section may be divided by subheadings. It should provide a concise and precise description of the experimental results and their interpretation, as well as the experimental conclusions that can be drawn.

## 4. Discussion

### 4.1. Highlights

In this retrospective study, we assessed the distinct epileptic network of different temporal lobe LEAT subtypes by analyzing voxel-wise metabolic pattern analysis. Our results show that the epileptic network of the amygdala–hippocampal LEAT involved is widespread, mainly including temporal neocortex and subcortical structures, while the network of the temporal neocortex LEAT is constrained, which is confirmed by the subgroup analysis. Furthermore, we performed the first SEEG-based analysis in brain tumors and observed distinct EZ organizations: (1) mesial temporal LEATs showed the complex organizations widely involved the nearby cortex and (2) temporal neocortex LEAT displayed the limited organizations primarily involving the perilesional cortex.

### 4.2. Metabolic Network of Temporal Lobe LEAT

PET plays a significant role in the presurgical workup in epilepsy surgery. The previous study has validated the utility of PET in localizing ictal foci in TLE patients, even in those with normal MRI [21]. In our study, the mesial temporal lobe LEAT represents more widespread hypometabolism and severe effects on the adjacent cortex, which is concordant with previous studies that showed the widespread ipsilateral hypometabolism in mTLE, involving temporal (mesial structures, pole, and lateral cortex) and extratemporal areas (insula, frontal lobe, perisylvian regions, and thalamus) [21,22,23]. The results in the present study mainly include the temporal neocortex, CN, putamen, and thalamus. Our previous cortico-cortical evoked potential (CCEP) study confirmed that the temporal neocortex had strong connectivity to the hippocampus in patients with TLE [24], which indicates that clinical ^18^FDG-PET findings are in concordance not only with EEG and MRI [23], but with intracranial electrophysiology as well. The function of CN in TLE was rarely reported previously, but a study calculating the functional connectivity (FC) of resting-state functional MRI (rs-fMRI) revealed that nucleus accumbens (NAc) serve vital roles for mTLE [25]. The putamen is considered as important in the generation of dystonic posturing, a reliable lateralizing symptom for mTLE, which was confirm by an ictal single-photon emission computed tomography (SPECT) research [26]. The thalamus is also considered a crucial hub in the pathological network of TLE [23,27]. The hypometabolism in the paracentral lobe is accordant with a previous article, which considered it to be related to the symptomatic generation and modulation of hand automatisms in mTLE [28].

In contrast, compared to the mesial temporal lobe LEAT, our results show lateral the temporal lobe LEAT represents the limited metabolic network, which mainly involved temporal neocortex, caudate nucleus, thalamus, and paracentral lobe. The hypometabolism range was similar to a previous article [29]. Considering the potential influence of the heterogeneity of tumor location and shape, we then performed the subgroup analysis that further divided the mesial temporal lobe LEAT group into the amygdala–hippocampal LEAT subgroup and the temporal base LEAT group to exclude the offset effect. The subgroup analysis with more homogeneity duplicated the above results and confirms the phenomenon that the metabolic pattern of the temporal neocortex LEAT is constrained.

### 4.3. SEEG Implantation and Epileptogenicity in Patients with Temporal Lobe LEAT

The relatively clear anatomo-electro-clinical profile and lesion border make SEEG implantation uncommon in patients with a brain tumor. Therefore, the intracranial electrophysiological evidence of the tumor is rare, and even the sample is small. In the present study, two patients underwent unconventional SEEG because of the “temporal plus epilepsy (TPE)” consideration. TPE is a major determinant of temporal lobe surgery failures. The most commonly affected neighboring regions include the insula (4.2%), the suprasylvian operculum (2.4%), the orbitofrontal cortex (1.2%), and the temporo-parieto-occipital junction (3.0%) [30]. Patient 64, with hippocampal GG, displayed autonomic symptoms (arrhythmia), increased FLAIR intensity, and hypometabolism in the left temporal and insular lobe. Patient 70, with temporal pole GG, displayed hypometabolism in the left insular lobe. Both of them finally underwent SEEG because of the consideration of the potential neurological deficit by operation in the dominant hemisphere and the atypical representation.

Given what we know, our study is the SEEG-based analysis for a brain tumor. The intracranial electrophysiological evidence of the temporal lobe LEAT is remarkably similar to the metabolic pattern. A study showed a gradient of the PET hypometabolism from non-involved to propagation, and then to epileptogenic and lesional zones [31]. In the present study, the epileptic activity involves more structures and a larger pathological network in patients with a mesial temporal LEAT. Meanwhile, a high epileptogenicity index is mainly focused in the lesional and perilesional cortex in patients with a lateral temporal lobe LEAT (Figures S1 and S2).

### 4.4. Surgical Indication for Tumor-Associated Temporal Lobe Epilepsy

For most cases, lesionectomy is preferred and achieves a satisfying prognosis, especially for tumors located in the neocortex. The previous study supported that lesionectomy alone for lateral temporal cavernous malformation (CM) can obtain excellent seizure outcomes [32]. Another MRI-based study classified DNT into three types, according to different MRI features, and recommended that resection should be restricted in type 1 (cystic/polycystic-like) but extensive in types 2 (nodular-like) and 3 (dysplastic-like) [33]. This offered a scheme for determining the extent of resection when planning surgery and obtaining the best functional results [34]. Meanwhile, ATL is a typical surgical approach for mTLE with hippocampal sclerosis (HS), but whether it is an optimal surgical strategy for mesial temporal lobe LEATs remains inconclusive. Considering the complex organization of the epileptic network and function protection, preferred surgical resection is worth exploring.

### 4.5. Limitation

Some limitations exist in the present study. To increase the statistical power, we flipped the PET images horizontally in patients with a left-sided temporal LEAT; however, brain networks might be different between the left and the right hemisphere. Another limitation is the small sample size of tumors with intracranial electric information. However, as we discussed above, these data are rare, as SEEG is not common for brain tumors. Accumulative cases are warranted to extend and confirm the findings in our study.

## 5. Conclusions

Our study demonstrates a more complex and widespread organization of the epileptic network, based on ^18^FDG-PET, in patients with a mesial temporal lobe LEAT than in those with a lateral temporal lobe LEAT. The SEEG-based epileptogenicity analysis further verifies this distinct metabolic pattern. A better understanding of the epileptic network is conducive to establishing individual surgical strategies for temporal lobe LEATs.

## Figures and Tables

**Table 1:** Patient demographic and clinical information.

	Patients with Mesial Temporal LEATs	Patients with Lateral Temporal LEATs	Healthy Controls	Statistic
Number	43	27	25	-
Sex (female, %)	20 (46.5%)	14 (51.9)	12 (48.0%)	Pearson *χ*^2^ = 0.192, *df* = 2, *p* = 0.909
Age of surgery (yrs)	20.1 (12.0)	23.9 (11.0)	26.6 (8.7) *	ANOVA *F* = 0.2.964, *df* = 2, *p* = 0.057
Duration (yrs)	5.7 (5.2)	7.6 (8.1)	-	Mann-Whitney *U* = 552.000, *df* = 1, *p* = 0.731
Age of onset (yrs)	14.4 (11.4)	16.3 (9.5)	-	Student’s *t* = −0.734, *df* = 1, *p* = 0.465
Side (left, %)	18 (41.9%)	13 (48.1%)	-	Pearson *χ*^2^ = 0.266, *df* = 1, *p* = 0.606
size of LEAT (mm^3^)	8253.0 (10748.6)	6195.3 (4163.9)	-	Mann-Whitney *U* = 501.5, *df* = 1, *p* = 0.340
Pathology (%)	GG: 33 (76.7%) DNT: 5 (11.6%) LGNETs: 2 (4.7%) AG: 1 (2.3%) PGNT: 1 (2.3%) PA: 1 (2.3%)	GG: 20 (74.1%) DNT: 5 (18.5%) AG: 1 (3.7%) LGNETs: 1 (3.7%)	-	-
Duration (months)	44.1 (16.0)	43.3 (15.0)	-	Mann-Whitney *U* = 555.500, *p* = 0.763

*: The time of PET scan for healthy controls; LEATs: long-term epilepsy-associated tumors; category data represented as a number (percentage) and continuous data represented as the mean (standard deviation); GG: ganglioglioma; DNT: the dysembryoplastic neuroepithelial tumor; AG: the angiocentric glioma; PGNT: the papillary glio-neuronal tumor; PA: the pilocytic astrocytoma; LGNETs: low-grade neuroepithelial tumors (not otherwise specified).

**Table 2:** Statistical analysis in ROI-based metabolic comparison among different groups.

	Test for Normal Distribution			Test of Homogeneity of Variances		Within-Group Comparison		Post *Hoc* Test				
	Groups	Lilliefors Statistic	*p* Value	Levene Statistic	*p* Value	Statistic	*p* Value	Multiple Comparisons	Contrast	MD	*p* Value	95% CI
PCL	Mesial	0.103	0.285	0.146	0.864	ANOVA *F*: 9.731	<0.001 *	Scheffe	mesial vs. lateral	−0.007	0.636	−0.024, 0.011
	Lateral	0.129	0.289						mesial vs. control	−0.031	<0.001 *	−0.048, −0.013
	Control	0.146	0.175						latera vs. control	−0.024	0.010 *	−0.043, −0.005
CN	Mesial	0.090	0.500	5.062	0.008 *	ANOVA *F*: 20.749	<0.001 *	Games-Howell	mesial vs. lateral	−0.015	0.017 *	−0.028, −0.002
	Lateral	0.122	0.355						mesial vs. control	−0.045	<0.001 *	−0.066, −0.024
	Control	0.090	0.500						latera vs. control	−0.030	0.004 *	−0.051, −0.009
Putamen	Mesial	0.140	0.034 *	3.245	0.043 *	Kruskal-Wallis *H*: 19.258	<0.001 *	Bonferroni	mesial vs. lateral	−14.417	0.084	−30.282, −1.448
	Lateral	0.114	0.472						mesial vs. control	−30.207	<0.001 *	−46.457, −13.956
	Control	0.152	0.136						latera vs. control	−15.790	0.098	−33.723, 2.144
Thalamus	Mesial	0.114	0.164	0.780	0.461	ANOVA *F*: 4.754	0.011 *	Scheffe	mesial vs. lateral	−0.033	0.140	−0.073, 0.008
	Lateral	0.149	0.123						mesial vs. control	−0.049	0.017 *	−0.090, −0.007
	Control	0.105	0.500						latera vs. control	−0.161	0.680	−0.062, 0.030

PCL: Paracentral lobule; CN: caudate nucleus; *: significant difference; MD: mean difference; 95% CI: 95% confidence interval.

## Data Availability

The data that support the findings of this study are available from the corresponding author upon reasonable request.

## Supplementary Materials

The following supporting information can be downloaded at: https://www.mdpi.com/article/10.3390/jcm11185309/s1, Figure S1: Interictal epileptiform and ictal epileptogenicity index (EI) for Patient 70 with lateral temporal lobe LEAT. (A) anatomical representation of LEAT and the SEEG implantation; (B) MRI-PET coregistration with an electrode through the lesion. The normalized PET value, spike and high frequency oscillation (HFO) counts were calculated in each contact; (C) Interictal oscillation maps of electrodes of amygdala, hippocampus, adjacent gyrus, distance, lesional and perilesional cortex; (D) Ictal EI map of electrodes of amygdala, hippocampus, adjacent gyrus, distance, lesional and perilesional cortex. The highest epileptogenicity was observed in lesional and perilesional cortex; Figure S2: Epileptogenicity index calculation of all recorded seizures of Patient 70. Ictal EI map of electrodes of amygdala, hippocampus, adjacent gyrus, distance, lesional and perilesional cortex. The highest epileptogenicity was observed in lesional and perilesional cortex. References [35,36] are cited in the supplementary materials.
