# Supplementary material for: Metabolism and Intracranial Epileptogenicity in Temporal Lobe Long-Term Epilepsy-Associated Tumor
Source: J Clin Med. 2022 Sep 9;11(18):5309. doi: 10.3390/jcm11185309 (PMC9504693; doi:10.3390/jcm11185309)
Supplement: Supplementary file 1 [file jcm-11-05309-s001.zip › jcm-1803506-supplementary.pdf]

Metabolism and intracranial epileptogenicity in temporal lobe long-term epilepsy-associated tumor

Supplemental material

Content S1: Method approach for epileptogenicity index calculation.

Figure S1. Interictal epileptiform and ictal epileptogenicity index for Patient 70 with lateral temporal lobe LEAT.

Figure S2. Epileptogenicity index calculation of all recorded seizures of Patient 70.

Content S1. Method approach for epileptogenicity index calculation

A baseline void of any epileptic activity or artifact was chosen during non-rapid eye movement sleep recording. All seizure episodes recorded by video monitoring and ictal SEEG patterns of electrical onset were identified visually in presurgical evaluation. The analysis was performed in a bipolar montage.

Interictal spikes and high-frequency oscillations (HFOs) were automatically detected using Delphos of AnyWave [1]. Delphos was design to detect events of interest above a threshold in the ZH10 normalized time-frequency images and measure the time width and frequency spread of the detected islands. Delphos classifies the detections as "spike" and "oscillation" with high precision and sensitivity across brain regions and across variations in brain activities [2]. For each channel, we computed the rate per minute for two included markers: spike and HFO. Before and after the detector was run, we visually verified the muscle artifact and high-frequency noise.

The ictal discharge of each seizure was quantitively calculated as the epileptogenicity index (EI) to define the seizure onset zone. EI ranks brain structures according to the tonicity of the fast discharge and the delay of involvement of the structure at seizure onset. Its values range from 0 to 1, with 1 corresponding to the most epileptogenic region. Channels exhibiting EI values above 0.3 are considered to be epileptogenic [3].

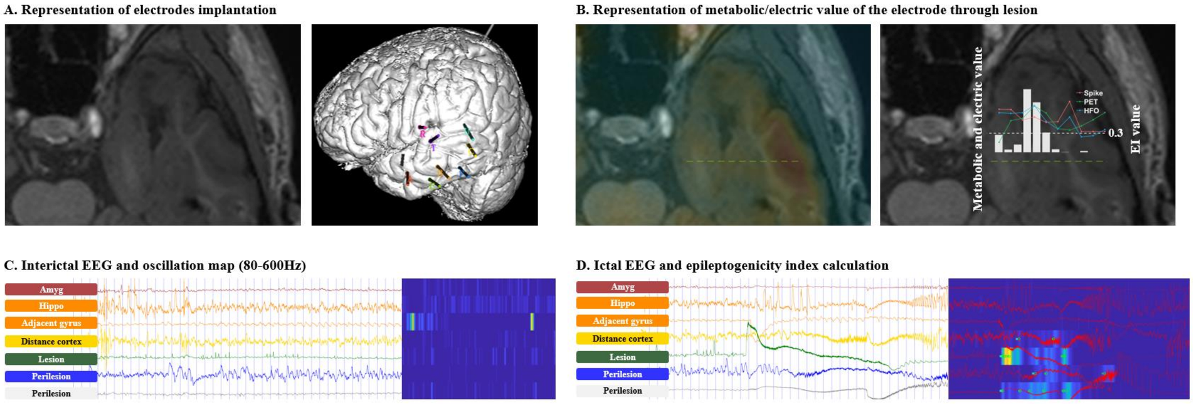

Figure S1. Interictal epileptiform and ictal epileptogenicity index (EI) for Patient 70 with lateral temporal lobe LEAT. (A) anatomical representation of LEAT and the SEEG implantation; (B) MRI-PET coregistration with an electrode through the lesion. The normalized PET value, spike and high frequency oscillation (HFO) counts were calculated in each contact; (C) Interictal oscillation maps of electrodes of amygdala, hippocampus, adjacent gyrus, distance, lesional and perilesional cortex; (D) Ictal EI map of electrodes of amygdala, hippocampus, adjacent gyrus, distance, lesional and perilesional cortex. The highest epileptogenicity was observed in lesional and perilesional cortex.

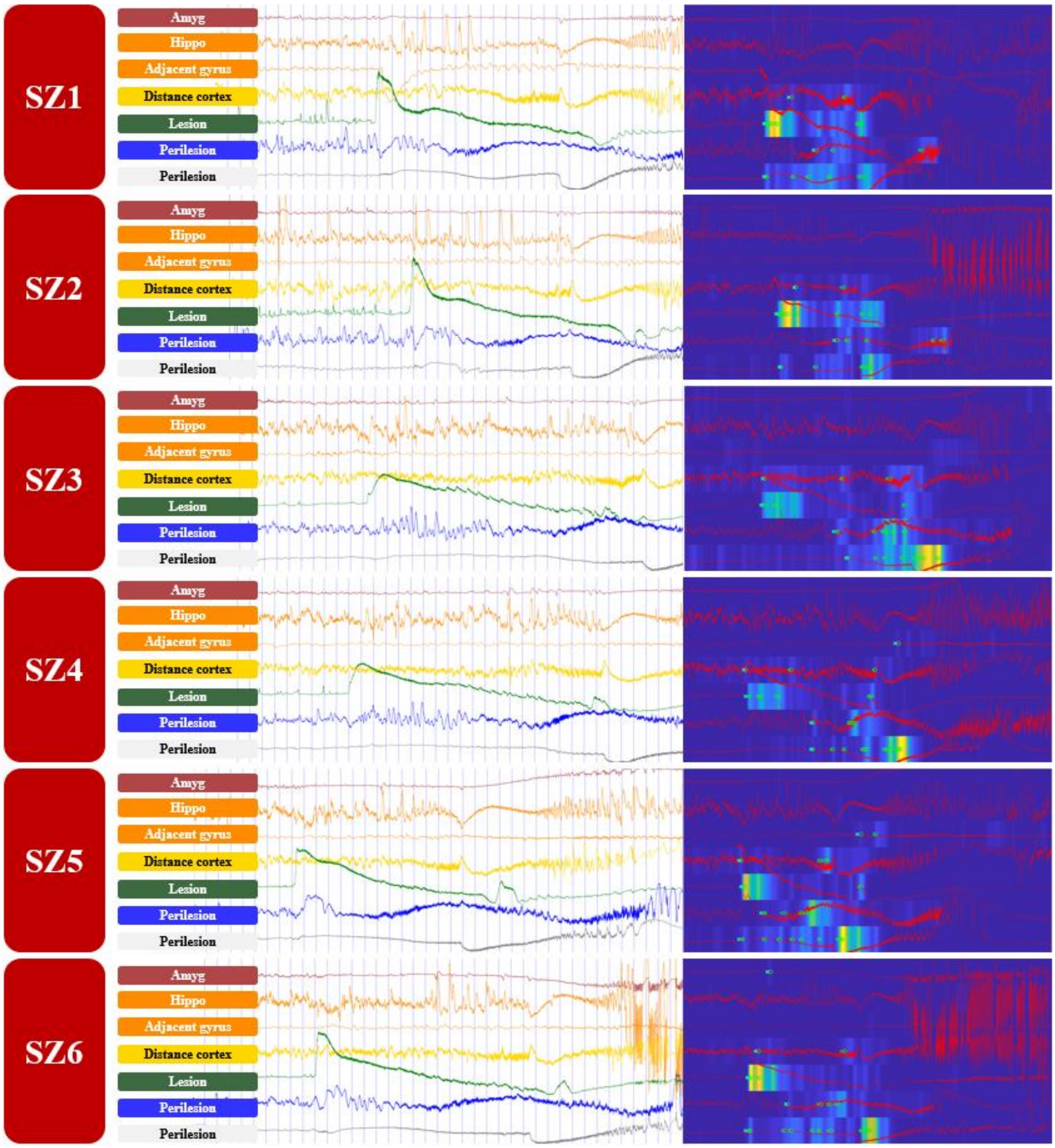

Figure S2. Epileptogenicity index calculation of all recorded seizures of Patient 70. Ictal EI map of electrodes of amygdala, hippocampus, adjacent gyrus, distance, lesional and perilesional cortex. The highest epileptogenicity was observed in lesional and perilesional cortex.

References

1. Roehri N, Lina J, Mosher J, Bartolomei F, Benar C: **Time-Frequency Strategies for Increasing High-Frequency Oscillation Detectability in Intracerebral EEG**. *IEEE transactions on bio-medical engineering* 2016, **63**(12):2595-2606.
2. Roehri N, Pizzo F, Lagarde S, Lambert I, Nica A, McGonigal A, Giusiano B, Bartolomei F, Bénar C: **High-frequency oscillations are not better biomarkers of epileptogenic tissues than spikes**. *Annals of neurology* 2018, **83**(1):84-97.

3. Bartolomei F, Chauvel P, Wendling F: **Epileptogenicity of brain structures in human temporal lobe epilepsy: a quantified study from intracerebral EEG**. *Brain : a journal of neurology* 2008, **131**:1818-1830.
